# Gestational diabetes in Germany—prevalence, trend during the past decade and utilization of follow-up care: an observational study

**DOI:** 10.1038/s41598-023-43382-6

**Published:** 2023-09-27

**Authors:** Veronika Lappe, Gregory Gordon Greiner, Ute Linnenkamp, Anja Viehmann, Heinke Adamczewski, Matthias Kaltheuner, Dietmar Weber, Ingrid Schubert, Andrea Icks

**Affiliations:** 1 PMV Research Group, Faculty of Medicine and University Hospital Cologne, University of Cologne, Herderstraße 52, 50931 Cologne, Germany; 2 Institute for Health Services Research and Health Economics, German Diabetes Center, Auf’m Hennekamp 65, 40225 Düsseldorf, Germany; 3 Institute for Health Services Research and Health Economics, Centre for Health and Society, Faculty of Medicine, Heinrich Heine University Düsseldorf, Moorenstr. 5, 40225 Düsseldorf, Germany; 4 WinDiab gGmbH, Geranienweg 7a, 41564 Kaarst, Germany; 5 German Center for Diabetes Research (DZD), Ingolstädter Landstraße 1, 85764 Munich-Neuherberg, Germany

**Keywords:** Gestational diabetes, Disease prevention, Epidemiology, Population screening, Risk factors

## Abstract

Gestational diabetes mellitus (GDM) is one of the most common medical complications in pregnancy. Information on key figures such as screening rates, prevalence of GDM or utilization of follow-up care and associated factors varies widely and is often lacking. The aim of our study is to provide information on screening rates for and prevalence of GDM as well as utilization of follow-up care in Germany. We used data (2010–2020) from a large, nationwide statutory health insurance containing information on inpatient and outpatient care, including diagnoses, medication and treatments. Descriptive analyses were performed to assess screening rates, prevalence of GDM and participation rates in follow-up care. A log-binomial regression model was calculated to analyze associated factors. Screening rates among pregnant women increased from 40.2% (2010) to 93.3% (2020) and prevalence from 9.4% (2010) to 15.1% (2020). The proportion of women attending follow-up care remained stable over time (around 42%). Age, educational level, insulin prescription, hypertension and obesity were positively associated with participation in follow-up care. Although over 90% of women in Germany are screened for GDM during pregnancy, follow-up care is used much less. Further research is needed to understand the trends in GDM healthcare (from screening to follow-up care) and the reasons for women's (non-)participation, as well as the attitudes and routines of the healthcare providers involved.

## Introduction

Gestational diabetes mellitus (GDM, ICD-10: O24.4G, International Statistical Classification of Diseases and Related Health Problems) is defined as a glucose intolerance diagnosed for the first time in pregnancy without reaching the diagnostic criteria for manifest diabetes^1^. Affected women have higher rates of adverse perinatal outcomes like caesarean section or shoulder dystocia^2–4^. Blood glucose levels often return to normal after childbirth, but this is strongly dependent on risk factors such as age, weight, or weight gain during pregnancy^5^. However, women who had GDM have an increased risk of developing Type 2 diabetes mellitus and cardiovascular diseases later in life, and a high risk of GDM recurring with further pregnancies^6–9^. In addition, a prospective cohort study reported, that offspring of mothers with GDM have an increased lifetime risk of overweight and obesity with concomitant cardiovascular and/or metabolic disorders^10^.

In light of the associated risks for mothers and their offspring after GDM, it is necessary to inform the women well and to recommend further health care services to minimize negative consequences of GDM. Reliable information on key figures of GDM health care is an important requirement to provide targeted support to women with GDM, like self-management programs^11^.

According to maternity guidelines in Germany, screening for GDM is offered to every pregnant woman who has not been diagnosed with diabetes before pregnancy or before 24th week of gestation between the 24th and 28th week of gestation with a two-step test procedure^12^. In Germany, information about the prevalence of GDM is rare and differs widely. Using perinatal data, the Institute for Quality Assurance and Transparency in Healthcare (IQTIG) estimated that 5.38% (40.845 of 758,614 pregnant women) in Germany in 2017 had GDM^13^, which increased to 7.55% (56,193 of 744,297 pregnant women) in 2020^14^. It is discussed that the real prevalence is underestimated, as the diagnosis may be insufficiently documented in the maternity records. A study using the nationwide billing data from the outpatient sector estimated a prevalence of 13.2% for GDM in Germany^15^. However, some methodological limitations have to be considered when interpreting the data, e.g. the assessment of eligible pregnant women was exclusively based on outpatient billing data and pregnant women had to use maternity care in at least 3 consecutive quarters to be included in the study. Data from statutory health insurance (SHI) can overcome these limitations.

In Germany, follow-up care for GDM is described in the maternity guidelines as well^12^. It is recommended that women with GDM receive an OGTT 6–12 weeks postpartum and regular follow-up examinations. However, previous regional studies suggest that only a limited proportion of women participate in postpartum testing^16,17^.

Therefore, the aim of this study was to provide information on key figures of GDM health care (screening rate, prevalence, proportion of woman attending postpartum screening and follow-up examinations after GDM) in Germany in the last decade (2010–2020) based on nationwide SHI data considering possible associated factors.

## Methods

### Data source

This observational study is based on data of a large, nationwide SHI with about 8.5 million insured persons. Data on inpatient and outpatient care were available as well as diagnoses coded via the International Statistical Classification of Diseases and Related Health Problems (ICD-10) and medication coded via the Anatomical Therapeutic Chemical Classification System (ATC). Treatments in hospitals are documented using German Diagnosis Related Groups (G-DRG) and OPS-Codes (Operation and Procedure Classification System, the German modification of the International Classification of Procedures in Medicine). In the outpatient sector physicians documented the treatment according to the German Uniform Value Scale (Einheitlicher Bewertungsmaßstab, EBM).

### Study sample

Women aged 13–49 years old who gave birth within a calendar year were selected via the non-changeable anonymous insurance number. Data of women giving birth in the years 2010–2020 were available. In Germany over 98% of the women give birth in a hospital^18^ and can be identified by the G-DRGs O01A to O01H for different types of caesarean section, and O02A with O02B, as well as O60A to O60D for vaginal delivery. In 130 cases more than one childbirth within one year was documented in the data set. In this case, the pregnancy preceding the first delivery in the year was included in the study. Length of pregnancy was identified by ICD-10-codes O09 that describe the length of pregnancy counted from the last menstruation summarising several weeks, e.g. O09.4 corresponds to gestational week 26 to 33. The ICD-Code coding the latest week of gestation less 20 days was taken to describe the length of pregnancy from conception to childbirth with O09.6 describing the normal length of pregnancy with 267 days. The date of childbirth is provided to the SHI but was not available in the data set for this study. Therefore, the first date with an OPS-Code or if missing the date of hospital admission of the hospital stay ending up in childbirth was taken as date of birth.

**Figure 1:**
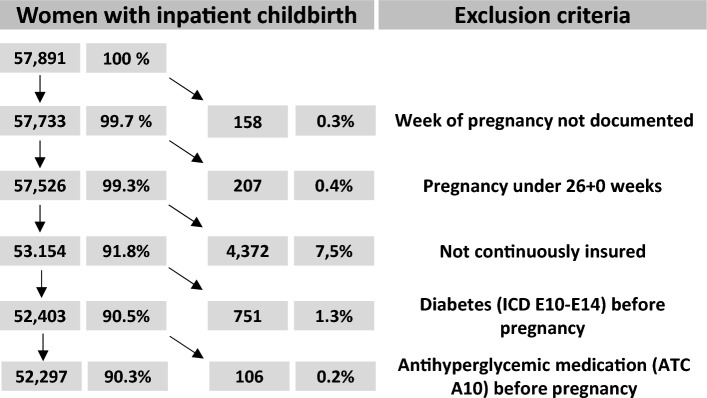
Flow-Chart of the study sample of women with inpatient childbirth exemplary for 2019.

We had to consider some exclusion criteria to reach our final study sample. Exemplary, this is shown in the Fig. 1 for the year 2019, as this is the most recent year for which the statistical analyses were fully performed. First, pregnancies without coded duration of pregnancy were excluded (0.3%). Secondly, the screening for GDM should be completed between week 24 and 28 of gestation. To include only women who had a chance to participate in the screening pregnancies ending prior to the 26^th^ week of gestation were excluded (0.4% of all pregnancies). To be able to exclude women with diabetes prior to pregnancy, women had to be continuously insured in the SHI four calendar quarters before the start of pregnancy until childbirth. In the cohort of women giving birth in 2019, 7.5% had to be excluded because they were not insured the whole time and 1.3% because of manifest diabetes before pregnancy. The latter—diabetes prior to pregnancy—was assumed when (i) ICD-codes E10 to E14 were verified in outpatient care during the four quarters before conception according to the quarterly documentation of outpatient diagnoses or (ii) an inpatient diagnosis was documented or (iii) a prescription of antidiabetic medication (ATC-Code A10) was issued in the prior 12-months before conception.

### Women with diagnosed GDM

Women with GDM were identified by at least one verified outpatient diagnosis ICD-10-code O24.4 in the calendar quarters from beginning of pregnancy to the quarter of childbirth. Inpatient diagnoses were included as well between conception and childbirth. The ICD-10-diagnoses O24.0, O24.1 and O24.3 classify pre-existing diabetes mellitus in pregnancy, Type 1, Type 2 diabetes mellitus and other, respectively. As they point to a pre-existing manifest diabetes, women with diagnosis O24.4 and one of these diagnoses (0.3% to 0.4% of women with childbirth) were not included as women with GDM to provide a conservative estimation of the GDM prevalence.

### GDM screening

Since the second half of the year 2013 in Germany a universal two-step screening for GDM is offered to pregnant women, which usually takes place in the 24-28th week of pregnancy. If the pre-test (50 g Glucose, non-fasting) results in abnormal blood glucose level (≥ 135 mg/dl (7,5 mmol/l)) after one hour, the OGTT (75 g Glucose, fasting) is performed on another day. GDM is diagnosed if any of the following blood glucose levels are exceeded: Fasting 92 mg/dl (5.1 mmol/l); after 1 h 180 mg/dl (10.0 mmol/l); after 2 h: 153 mg/dl (8.5 mmol/l). The screening is paid by the SHI and the EBM codes, i.e. 01776 (named “Pre-test for gestational diabetes”), 01777 (named “OGTT”), and 01812 (named “Glucose determination (screening for gestational diabetes)”, are documented in the data. To cover blood glucose monitoring before onset and beyond of screening the EBM Codes 32025 and 32057 (quantitative determination of glucose), 32881 (laboratory flat charge/fasting plasma glucose) and 32094 (quantitative determination of HbA1 or HbA1c) were included in analysis.

### GDM follow-up care

In Germany, women with GDM are recommended to receive an OGTT (75 g Glucose, fasting) 6 to 12 weeks postpartum to diagnose diabetes mellitus (fasting plasma glucose > 126 mg/dl (> 7.0 mmol/l) and/or OGTT-2-h value > 200 mg/dl (> 11.1 mmol/l)), IFG (impaired fasting glucose, fasting plasma glucose of 100–125 mg/dl (5.6–6.9 mmol/l), or IGT (impaired glucose tolerance, 2-h plasma glucose in the OGTT in the range 140–199 mg/dl (7.8–11.0 mmol/l)) ^19^. As there is no special EBM-Code for this OGTT postpartum two-times measurements of blood glucose were considered as an OGTT. As in the screening the EBM Codes 32025, 32057, 32881, and 32094 were included in analysis of postpartum blood glucose monitoring as well as the codes 01776, 01777, and 01812 (available for use since 2013) to cover testing in the context of a subsequent pregnancy. The follow-up care rates are based on women with diagnosed GDM continually insured 365 days after childbirth.

### Possibly associated factors

To consider risk differences in pregnancies we performed our statistical analyses after categorizing into three age groups used for descriptive analyses as well: younger pregnant women aged 13 to 25 years, older pregnant women aged 36–49 years (in Germany classified as high-risk pregnancy) and as reference group the middle-aged pregnant women 26–35 years old. For education level we used the highest vocational training of the employee that the employer communicates to the SHI whether or not it is essential for the current work. Vocational training was divided into the following five categories: unknown, without vocational training, officially accredited vocational training (reference group), higher vocational training like master craftsman, technician, equivalent technical college degree or Bachelor and highest vocational training referring to a University degree equivalent to a Master or higher. Prescription of insulin during pregnancy versus no prescription was identified by the ATC-code A10A. Obesity (ICD-Code E66) and hypertensive disorder (I10 to I15) prior pregnancy were considered as risk factors associated with the development of diabetes. Diagnosis versus no diagnosis in the four quarters before pregnancy were compared. In outpatient care verified diagnoses were considered as well as the discharge diagnosis and secondary diagnoses documented in inpatient care.

### Statistical analyses

We performed descriptive analyses to assess the screening rates for GDM, the prevalence of GDM, meaning identified and documented GDM diagnosis visible in the SHI data, and the attendance rates of GDM follow-up care.

The percentage of women with a performed screening was based on all women with childbirth in our final study sample. GDM prevalence was calculated as percentage of women with diagnosed GDM related to the cohort of women with childbirth in the respective calendar year in total and age-stratified. The attendance rate of GDM follow-up care was based on all women who received postpartum blood glucose controls in the year after childbirth related to all women with diagnosed GDM and continually insured 365 days after childbirth.

To analyze associated factors named above that may influence the attendance of GDM follow-up care we calculated a log binominal regression model.

To analyze data the SQL server Microsoft SQL Server Management Studio v18.7.1 was used and to calculate the statistical models SAS Enterprise Guide 8.5 (SAS Institute Inc., Carry, NC, USA) was used. Level of significance was α = 0.05.

### Ethics approval

This is an observational, retrospective study based on pseudo-anonymized claims data conducted in accordance with the principles of the Declaration of Helsinki. Data was used and analyzed in accordance with the legal provisions (§ 75 SGB X, Social administrative procedures and social data protection, https://www.sozialgesetzbuch-sgb.de/sgbx/75.html) that regulates the use of claims data and in accordance with the Guideline “Good Practice in Secondary Data Analysis”—in compliance with all data protection regulations. No experiments with humans or human tissue samples were performed. No other data were collected as part of the study.

## Results

### Cohorts of women with childbirth

Between 2010 and 2020, yearly about 55,000 women insured by the SHI gave birth at a hospital with pregnancy duration of 25 weeks or more and no manifest diabetes before pregnancy. During these years the proportion of women with childbirth aged 26–35 years remained stable around 67%. The oldest age group 36–49 years grew from 19.7 to 24.7% while the youngest age group 13–25 years got smaller from 14.7 to 9.3%.

### Screening for GDM and prevalence of GDM

**Figure 2:**
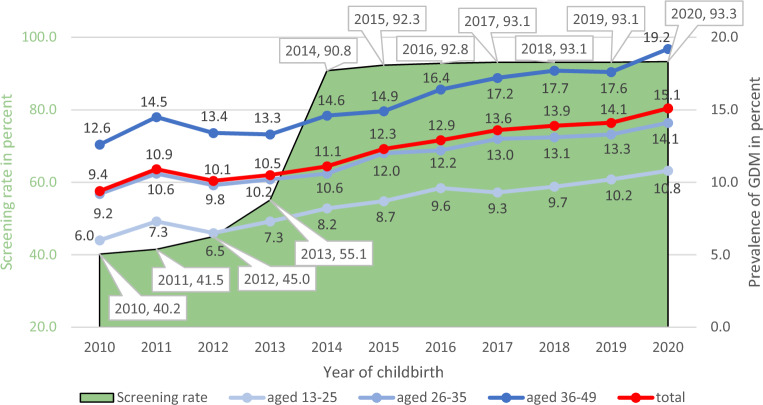
Screening rate and prevalence of gestational diabetes (GDM) in cohorts of women delivering 2010 to 2020*.* Number of women with childbirth in 2010 n = 55,328, 2011 n = 54,174, 2012 n = 56,023, 2013 n = 57,458, 2014 n = 59,179, 2015 n = 58,013, 2016 n = 57,763, 2017 n = 56,771, 2018 n = 55,164, 2019 n = 52,297, 2020 n = 51,038. Figures in boxes = screening rate.

In 2010, before introduction of the mandatory GDM screening, 40.2% of pregnant women were tested for impaired glucose. After implementation of the universal GDM screening in the second half of 2013, 2014 was the first year in which all women giving birth could completely take advantage of the implementation. The proportion of screened women rose from 85.3% in 2014 to 89.8% in 2020. Furthermore, some women had measurements of blood glucose, determination of HbA1 or HbA1c or laboratory flat charge (EBM Codes 32025, 32057, 32881, 32094) resulting in 90.8% tested for (gestational) diabetes in 2014 rising to 93.3% in 2020 (Fig. 2). About 10% of women are screened before the 24th week of pregnancy (mostly because of existing risk factors), about 70% in the scheduled period between the 24th and 28th week, and less than 1% after the 32nd week.

The prevalence of GDM rose from 9.4% (2010) to 15.1% (2020) over all age groups and was considerably higher in the oldest age group (Fig. 2).

### Insulin treatment

In 2020, 18.6% of women with GDM were treated with insulin during pregnancy. The proportion rose from 2010 over time not only in total from 14.4%, but especially in the oldest age-group (36–49 years) from 18.7 to 23.1% compared to an increase from 8.4 to 11.3% in the youngest age-group (13–25 years) and from 13.6 to 17.1% in women aged 26–35 years.

### Attendance of GDM follow-up care

**Figure 3:**
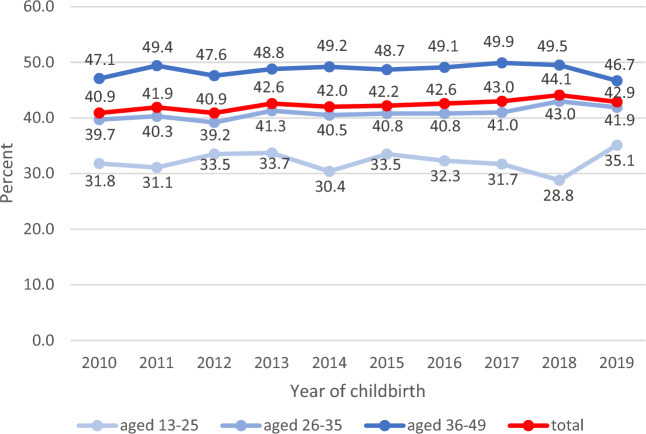
Attendance of GDM follow-up care in cohorts of women delivering 2010 to 2019.

Follow-up care of women with diagnosed GDM was analysed for one year after childbirth in the years 2010 to 2019. The proportion of women who received testing of blood glucose, HbA1/HbA1c or OGTT remained constant over time at about 42% (Fig. 3). Younger women up to 25 years with diagnosed GDM were tested only in about 32% postpartum, whereas women of older age (36–49 years) were tested in nearly 50% of the cases.

It is recommended to test women 6 to 12 weeks after childbirth ^1^. Only about 46% of women who were tested in the year after childbirth in the study period were tested within these first three months (minimum 2013 44.5%, maximum 2019 49.6%). Around 15% of the tested women were tested in the fourth month after childbirth.

### Factors associated with the attendance of GDM follow-up care

The results of the log binominal regression models analysing factors associated with the attendance of follow-up care in women with GDM within the first year after childbirth in 2019 are shown in Table 1. The mean age of this cohort was 32.9 years. Older women aged 36 years and above had a 6% (RR = 1.06, 95%-CI 1.01–1.12) higher probability for attendance of follow-up care compared to middle aged women and younger women up to 25 years of age had an 8% (RR = 0.92, 95%-CI 0.81–1.04) not significant lower probability. Women with higher level of education had an 12% higher probability to attend follow-up care compared to women with accredited vocational training reaching significance in the highest education level (RR = 1.12, 95%-CI 1.04–1.20). On the other hand, a lower level of education (women without vocational training) resulted in a 10% lower not significant probability to attend follow-up care. Insulin therapy during pregnancy raised the probability for attendance of follow-up care substantially (RR = 1.67, 95%-CI 1.58–1.76). Women with diagnosed obesity (RR = 1.17, 95%-CI 1.10–1.24) and hypertension (RR = 1.14, 95%-CI 1.05–1.23) had an around 15% higher probability for attendance of follow-up care than women without these diagnoses.

**Table 1:** Attendance of follow-up care during the year after childbirth in women with GDM delivering in 2019.

Factors investigated	Postpartum screening		RR^a^	95%-CI
	Yes (n = 3,045)	No (n = 4,060)		
Age (ref = Middle (26 to 35 years) n = 4,453)				
Younger (13 to 25 years) (%)	5.6 (n = 170)	7.8 (n = 315)	0.92	0.81–1.04
Older (36 to 49 years) (%)	33.2 (n = 1,011)	28.5 (n = 1,156)	1.06	1.01–1.12*
Education (ref = accredited vocational training n = 3,116)				
Unknown (%)	28.9 (n = 881)	30.2 (n = 1,227)	0.97	0.91–1.03
Without vocational training (%)	5.6 (n = 170)	7.1 (n = 288)	0.90	0.80–1.02
Master craftsman/technician/equivalent technical college degree or Bachelor (%)	6.7 (n = 205)	6.6 (n = 268)	1.10	0.99–1.22
Diploma/Magister/Master/State Examination or Doctorate/Habilitation (%)	14.7 (n = 446)	12.4 (n = 504)	1.12	1.04–1.20*
Prescription of insulin during pregnancy (ATC-Code A10A) (ref = no)				
Yes (%)	27.1 (n = 825)	10.5 (n = 425)	1.67	1.58–1.76*
Hypertension (ICD-Code I10-I15) (ref = no)				
Yes (%)	8.8 (n = 269)	5.4 (n = 220)	1.14	1.05–1.23*
Obesity (ICD-Code E66) (ref = no)				
Yes (%)	17.9 (n = 546)	11.2 (n = 456)	1.17	1.10–1.24*

^a^Estimation of associations of certain factors on the probability of performing a blood glucose test (EBM 32025, 32057, 32094, 32881) in the year after childbirth: Relative risk (RR) with 95% confidence interval (CI) from the log-binomial regression model adjusted for all listed variables; * significant (α = 0.05); women with childbirth in 2019 and gestational diabetes continuously insured in the year after childbirth (N = 7,105).

## Discussion

It can be stated that the start of pregnancy can be operationalized based on information on childbirth in SHI data and thus the time at which screening is used can be shown. We found a rise in the rate of measurements of blood glucose from 40.2% in 2010 before implementation of the structured screening program up to 90.8% in 2014 after implementation and 93.3% in 2020. Meanwhile, the prevalence of diagnosed GDM increased from 9.4% (2010) to 15.1% (2020). The proportion of women who attended follow-up care remained stable over time at about 42%. Age, educational level, prescription of insulin, hypertension and obesity were positively associated with the attendance of follow-up care and also significantly with age of the women.

To the best of our knowledge, this is the first study in Germany examining pregnant women from screening for GDM to follow-up care on SHI data.

A major strength of our study is that our analyses are based on data from one of Germany's largest SHI, covering a long period of time. The results represent the reality of care for a large population of insured women over several years, although it may be noted that there are, for example, slight age or socioeconomic differences from populations of other SHI or from data based on maternity logs. Besides, further strengths are lack of drop out, no recall-bias and no selection bias.

As a limitation, miscoding cannot be clearly excluded. For example, the diagnosis of GDM listed as a confirmed diagnosis could refer to a previous pregnancy. Even though it is assumed that GDM recurrence in more than 50%, partially up to 84% of women with previous GDM, it cannot be ruled out that a misclassification has occurred^8,9,20^.

Another limitation regarding the completeness of the screening, even though it reached 93.1%, could be that blood glucose measurements during inpatient stays are not included in the data and are therefore unknown. Before delivery, about 20 percent of the women were hospitalized during pregnancy.

A further limitation could be seen in the limited number of factors available to analyze the possible enabling or inhibiting influence for the use of postpartum screening. For example, we cannot draw information regarding lifestyle factors or number of previous pregnancies (with and without GDM) from the SHI data. We also do not have information on the health awareness of the women or information on the age and the attitudes and routines of the physicians towards postpartum screening. For example, the specialist group as well as the doctor-patient relationship could have a great influence on the utilization of the postpartum screening.

At present, the only official data available for prevalence of GDM in Germany are those in the reports Quality Assurance in Obstetrics published by the Institute for Quality Assurance and Transparency in Health Care^13,14^. The data is based on the documentation of GDM in the maternity log and used in other publications as well^21^. Compared to our study, which calculated a prevalence of 15.1% in 2020, the prevalence is clearly lower (2020: 7.55% of pregnant women)^14^. It is suspected, that GDM in the maternity log is documented insufficiently and that the prevalence is underestimated.

In our study, the prevalence of diagnosed GDM increased from 9.4% (2010) to 15.1% (2020) and, as expected, also significantly with age of the women. From 2014 to 2020 (after implementation of the screening program), a 2.5%-point increase (90.8–93.3%) of women with screening led to a 4.0%-point increase (11.1–15.1%) of diagnosed GDM. If one considers the period before the introduction of the mandatory screening, the proportion of women with GDM screening has more than doubled from 2012 till 2014 (45–91%), whereas the prevalence of GDM increased by 10% (from 10.1% in 2012 to 11,1% in 2014). The largest percentage increase was recorded in the youngest (and smallest) age group. It appears that physicians were already testing high-risk cases before implementation of the screening program, although we cannot analyse this due to limitations in observable variables (e.g. no information on other risk factors such as family history of diabetes, GDM in former pregnancy, weight gain during pregnancy, laboratory parameters or lifestyle factors). The increase in documented GDM diagnoses may have been associated with higher screening rates and greater physician awareness; however, increasing age at birth and an increase in risk factors (e.g. obesity) in general may play a bigger role ^22,23^. Internationally, the reported prevalence of GDM varies widely between regions and countries due to the lack of uniform screening approaches, differences in diagnostic criteria and different characteristics of the cohorts studied ^24^. The prevalence of GDM is thought to range from 1.8 to 25.4%, and up to 38.6% in obese pregnant women ^24–26^. A prevalence of around 15% seems reasonable in comparison with comparable countries. The prevalence of diagnosed GDM, similar to Type 2 diabetes mellitus, has shown an increasing time trend in recent decades and is expected to continue to rise in Europe as well as in the US ^27–32^. The true trend and GDM prevalence itself can only be determined by screening rates remaining approximately constant at a high level over the years.

In our study, across all age groups, although recommended in the guidelines, little more than 40% of women with GDM had at least one blood glucose check during a year after childbirth. This is in line with the numbers stated by the GestDiab register. The GestDiab register is the largest register in Germany that systematically collects data on diabetes and pregnancy with a focus in the densely populated North Rhine region ^33,34^. However, compared to international studies, the proportion of women receiving postpartum screening and attending follow-up care in Germany is at best moderate ^35–38^. Older women were more likely attending follow-up care than younger women. This may be due to greater awareness among women and health care providers and applied risk-adapted testing as the risk of developing manifest diabetes increases with age. Women with high educational status attended follow-up care more often significantly. There is a weak trend in our data that women with lower educational status tend to be less likely to seek follow-up care for GDM. Similar results were found by Tovar et al. ^39^.

The vast majority of all pregnant women in Germany participates in screening for GDM, but follow-up diabetes screening is only taken up by about 40% of those diagnosed with GDM. Reasons could be that pregnant women are in regular medical care during pregnancy due to preventive care. Pregnant women are committed to ensuring that their pregnancy goes well and that the child does well ^38^. After the birth of the child, mothers seem to have other priorities than their own health. Another study found that the experience of women undergoing OGTT was often associated with high levels of anxiety and mild to moderate physical pain ^40^. Based on the experience gained from screening, this could lower repeat participation in an OGTT among affected women. In addition, after pregnancy, health care provision often switches back from the gynecologist to the general practitioner ^41^. Further research is needed to understand the impact and consequences of involving various professional groups with potentially different experience and knowledge in the care of women with GDM.

## Data Availability

The datasets generated during and/or analysed during the current study are not publicly available due to data protection regulations for data of statutory health insurance but are available upon justified request in agreement with the SHI and upon successful application to the competent supervisory authority.
